# The rs11515 Polymorphism Is More Frequent and Associated With Aggressive Breast Tumors with Increased *ANRIL* and Decreased *p16*^*INK4a*^ Expression

**DOI:** 10.3389/fonc.2015.00306

**Published:** 2016-01-21

**Authors:** Janice A. Royds, Anna P. Pilbrow, Antonio Ahn, Helen R. Morrin, Chris Frampton, I. Alasdair Russell, Christine S. Moravec, Wendy E. Sweet, W. H. Wilson Tang, Margaret J. Currie, Noelyn A. Hung, Tania L. Slatter

**Affiliations:** ^1^Department of Pathology, Dunedin School of Medicine, University of Otago, Dunedin, New Zealand; ^2^Department of Medicine, University of Otago, Christchurch, New Zealand; ^3^Department of Pathology, University of Otago, Christchurch, New Zealand; ^4^Cancer Research UK Cambridge Institute, University of Cambridge, Cambridge, UK; ^5^Department of Cardiovascular Medicine, Kaufman Center for Heart Failure, Cleveland Clinic, Cleveland, OH, USA

**Keywords:** breast cancer, ANRIL, p16^INK4a^, 9p21, single-nucleotide polymorphisms, rs11515

## Abstract

Chromosome position 9p21 encodes three-tumor suppressors p16^INK4a^, p14^ARF^, and p15^INK4b^ and the long non-coding RNA *ANRIL* (antisense non-coding RNA in the INK4 locus). The rs11515 single-nucleotide polymorphism in the *p16^*INK4a*^*/p14*^ARF^* 3′-untranslated region is associated with glioblastoma, melanoma, and other cancers. This study investigated the frequency and effect of rs11515 genotypes in breast cancer. Genomic DNA samples from 400 women (200 with and 200 without a diagnosis of breast cancer) were genotyped for the rs11515 major (C) and minor (G) alleles. The rs11515 polymorphism was also investigated in 108 heart tissues to test for tissue-specific effects. Four 9p21 transcripts, *p16*^*INK4a*^, *p14^*ARF*^*, *p15^*INK4b*^*, and *ANRIL* were measured in breast tumors and myocardium using quantitative PCR. Heterozygotes (CG genotype) were more frequent in women with breast cancer compared to the control population (*P* = 0.0039). In those with breast cancer, the CG genotype was associated with an older age (*P* = 0.016) and increased lymph node involvement (*P* = 0.007) compared to homozygotes for the major allele (CC genotype). In breast tumors, the CG genotype had higher *ANRIL* (*P* = 0.031) and lower *p16^*INK4a*^* (*P* = 0.006) expression compared to the CC genotype. The CG genotype was not associated with altered 9p21 transcripts in heart tissue. In breast cancer, the rs11515 CG genotype is more frequent and associated with a more aggressive tumor that could be due to increased *ANRIL* and reduced *p16^*INK4a*^* expression. The absence of association between rs11515 genotypes and 9p21 transcripts in heart tissue suggests this polymorphism has tissue- or disease-specific functions.

## Introduction

Single-nucleotide polymorphisms (SNPs) within the 9p21 locus are associated with coronary artery disease (1–3) and multiple cancer types (4–7), suggesting this region is significant toward disease susceptibility. Three proteins encoded at 9p21 are well-defined tumor suppressors. The p16^INK4a^ and p15^INK4b^ proteins limit cell proliferation by inhibiting cyclin-dependent kinases, and the third tumor suppressor (p14^ARF^) prevents the degradation of p53 [reviewed in Ref. (8, 9)]. Different open-reading frames of the cyclin dependent kinase 2A (*CDKN2A)* gene encode p16^INK4a^ and p14^ARF^, while p15^INK4b^ is encoded by a separate gene (*CDKN2B*).

The rs11515 polymorphism in the *CDKN2A* 3′-untranslated region (UTR) consists of a major (C) and minor (G) allele at cDNA nucleotide 500 (numbered from the *p16^*INK4a*^* initiation codon). It has been associated with melanoma, sporadic colorectal, skin, bladder, and cervical cancers (10–17). In glioblastoma, the rs11515 G allele was associated with a worse prognosis and older age (17). This polymorphism is 5′ to the long non-coding RNA *ANRIL* (antisense non-coding RNA in the INK4 locus, also known as *CDKN2B-AS1*) (18). Increased *ANRIL* is associated with increased proliferation, metabolic activity, inflammation, and attenuation of apoptosis (19–22). Silencing of *ANRIL* prevented fibroblast and smooth muscle cell proliferation (19, 20) as *ANRIL* is increased as part of a bacterial response *ANRIL* may function as part of an inflammatory response (22). This and other evidence, including the findings of increased *ANRIL* in prostate cancer, leukemia, glioma, and breast cancer suggest *ANRIL*, may promote tumorigenesis (19, 23–26).

Given the association of the *CDKN2A* rs11515 and the functions of proteins encoded at 9p21 in cancer, the current study investigated rs11515 genotypes in breast cancer.

## Materials and Methods

### Patients

For the breast cancer analyses, the patient cohort consisted of 200 women diagnosed with primary breast cancer who underwent surgery at Christchurch hospital, New Zealand. The mean age for the breast cancer cohort was 59.7 years (95% CI 57.6–61.8). Ethnicity data were available for 152 women (87% identified as European, 3% Maori, 3% Maori and European, and 7% identified with other ethnic groups). The control cohort consisted of 200 healthy women from the New Zealand population. The mean age of the control cohort was 56 years (95 CI percentile 54–58.5 years). Ethnicity data were available for all in the control cohort. Eighty-five percent identified as European, 3% Maori, 5% Maori and European, and 7% identified with other ethnic groups. There was no significant difference in age between the patient and control cohorts. Frozen breast tumors were available for 25 women (12 women with the CG and 13 with the CC genotype). Tumor tissue was limited for four individuals with the CG genotype; therefore, some analyses were performed on 8 instead of 12 tumors. The breast tumors were selected so that four variables were matched between those with the CG and CC genotypes (tumor grade, presence of lymph/vascular invasion, ≥2 lymph nodes affected, and an estrogen-receptor positive tumor). All breast tumors were from women who did not have previous treatment with radiotherapy or chemotherapy before surgery.

For the analysis of heart tissue, tissue from the left ventricular free wall of the myocardium of organ donors (*n* = 108) was collected by the Cleveland Clinic Kaufman Center for the Heart Failure Human Heart Tissue Bank between 1993 and 2006, as previously described (27).

For the breast tumor study, ethical approval was obtained in New Zealand, and all women gave written informed consent for inclusion in the study (Ethics Committee Approvals 02.06.98/5.11.09 and LRS/10/09/035). Standard procedures were followed, which included culturally appropriate tissue handling and disposal protocols (28). For the heart tissue study, ethical approval was obtained from the Cleveland Clinic Internal Review Board (IRB 2378), and the study adhered to the principles outlined in the Declaration of Helsinki. All procedures followed were in accordance with institutional guidelines and all families and/or patients provided informed consent.

### Rs11515 Genotyping

The C and G rs11515 alleles were genotyped in the breast tumor and control cohorts using a PCR and restriction enzyme digestion method. Genomic DNA was extracted from blood leukocytes using the Dneasy Blood & Tissue Kit (Qiagen, Veno, Netherlands) according to the manufacturer’s instructions. A 319 bp region was amplified using the following primers: forward, 5′-TGCCACACATCTTTGACCTC-3′; reverse, 5′-GCAGAAGCGGTGTTTTTCTT-3′. Following conformation of the correct PCR product, amplified DNA was digested with the *Msp*I restriction enzyme. Restriction enzyme-digested PCR products were separated by agarose gel electrophoresis to identify the rs11515 genotype (C allele, digested band; and G allele, undigested band). DNA sequencing was performed on 20 PCR products to confirm genotypes as described previously (17).

The C500 and G500 rs11515 alleles were genotyped in heart tissue using a Taqman SNP assay (C_12096259_10, Applied Biosystems, Foster City, CA, USA). Genomic DNA was extracted from frozen tissue as previously described (27) and from samples genotyped in duplicate using the Lightcycler 480 platform and Lightcycler 480 software, version 1.5.0 (Roche, Indianapolis, IN, USA). Reactions were optimized for 5 μL volumes with 0.5× the recommended probe concentration.

### Rs3088440 Genotyping

For the breast tumor study, rs3088440 alleles were genotyped in the patient and control cohorts using a PCR and restriction enzyme digestion method. A 180 bp region was amplified from blood leukocyte genomic DNA using the following primers: forward, 5′-TAGATCATCAGTCACCGAAGG-3′; reverse, 5′-CATTTACGGTAGTGGGGGAAG-3′. Following conformation of the correct PCR product, amplified DNA was digested with the *Hae*III restriction enzyme. Restriction enzyme-digested PCR products were separated by agarose gel electrophoresis to identify the major allele (digested band) and the minor allele (undigested band). DNA sequencing was performed on 20 PCR products to confirm genotypes as described previously (17).

### *CDKN2A* Gene Dosage

The gene dosage of *CDKN2A*, exon 1α (*p16^*INK4a*^*), and exon 1β (*p14^*ARF*^*) was estimated in 13 breast tumors with the CC and 12 tumors with the CG rs11515 genotype using a multiplex PCR assay, as previously described (29). Tumor DNA was extracted using the Qiagen Dneasy Blood & Tissue Kit according to the manufacturer’s instructions (Qiagen, Limburg, The Netherlands). The intensity of the *CDKN2A* and **β*-globin* PCR products was compared using Bio-Rad Quantity One software (Bio-Rad Laboratories, CA, USA) and Syngene GeneTools image software (Syngene, Cambridge, UK) as previously described (17).

### Quantitative Real-Time PCR

For the breast tumor study, quantitative real-time PCR (RT-qPCR) was performed for *CDKN2A/p16^*INK4a*^*, *CDKN2A/p14^*ARF*^*, *CDKN2B*, and *ANRIL*. RNA was extracted from frozen tissue using the Ambion Purelink RNA Mini Kit (Life Technologies, Carlsbad, CA, USA) and tissue was homogenized (gentleMACS dissociator gentleMACs, Miltenyi Biotec, GmbH, Germany) according to the manufacturers’ instructions. Quantitative RT-qPCR was performed using Taqman Gene Expression assays with inventoried probes (Applied Biosystems, ThermoFisher Scientific, Waltham, MA, USA) for *CDKN2A/p16^*INK4a*^* (assay id: Hs02902543_mH), *CDKN2A/p14^*ARF*^* (assay id: Hs99999189_m1), *CDKN2B* (assay id: Hs00793225_m1) and *ANRIL* (assay id: Hs01390879_m1). Reactions were performed in duplicate on a Lightcycler 480 platform (Roche, Basel, Switzerland) and analyzed with Lightcycler 480 software, version 1.5.0 (Roche, Basel, Switzerland). Expression levels were converted to relative quantities and normalized to tumor protein, translationally controlled 1 (*TPT1*, assay id Hs02621289_g1) and eukaryotic elongation factor 1A1 (*EEF1A1*, assay id Hs00265885_g1), two reference genes previously validated for normalization of RT-qPCR data in breast and cancer cell types (30, 31).

For the study of heart donors, expression data for *CDKN2A/p16^*INK4a*^* and CDKN2A*/p14^*ARF*^* was obtained in the current study, and that for *CDKN2B* and *ANRIL* generated as part of a previous study (27) and included in the current analysis. RT-qPCR was performed using Taqman Gene Expression assays for *CDKN2A/p16^*INK4a*^* (assay id: Hs02902543_mH), *CDKN2A/p14^*ARF*^* (assay id: Hs99999189_m1), *CDKN2B* (assay id: Hs00793225_m1), and *ANRIL* (assay id: Hs01390879_m1) as described above. Expression levels were normalized to *TPT1* (assay id Hs02621289_g1), *EEF1A1* (assay id Hs00265885_g1) and signal recognition particle 14 kDa (*SRP14*, assay id Hs03055045_g1), three reference genes previously validated for use in human myocardium (32).

### Statistical Analyses

The genotype frequencies were compared between cases and controls using the Chi-square test and SHEsis software (33). For breast tumors and heart samples, clinicopathologic measures were compared between genotypes using Chi-square and one-way ANOVA followed by pairwise comparisons tests. All gene expression levels displayed skewed distributions and were log_e_-transformed prior to analysis. Associations between gene expression and rs11515 genotypes were performed with analysis of variance (genotypic model), with adjustment for age, as appropriate. Correlations in expression levels between genes were tested with Pearson correlation. Statistical analyses were performed with SPSS version 22 software and *P* < 0.05 was taken as a significant difference.

## Results

### The rs11515 Minor Allele Was More Frequent in Women with Breast Cancer

The rs11515 alleles were genotyped in woman with breast cancer and healthy women. In women with breast cancer, 61% (*n* = 121) were homozygote for the major allele (CC, genotype), 38% (*n* = 76) were heterozygote (CG, genotype), and 1.5% (*n* = 3) were homozygote for the minor allele (GG, genotype). The genotypes of 20 samples were confirmed using DNA sequencing (7 typed as CC, 10 typed as CG, and 3 typed as GG). In the control population, 76% (*n* = 152) had the CC, 23% had the CG (*n* = 46), and 1% of women had the GG genotype. The genotype frequencies in controls were in Hardy–Weinberg equilibrium (*P* = 0.47). The CG genotype was more frequent in women with breast cancer compared to the control population (*P* = 0.0039).

Clinicopathologic variables (patient age at presentation, tumor size, the number of nodes involved, venous and lymphatic invasion, estrogen receptor, progesterone receptor, and human epidermal growth factor 2 receptor statuses) among rs11515 genotypes are shown in Table 1 and were compared between genotypes in the breast cancer cohort. The mean age for those with the CG allele was higher (63.3 years, 95% CI 60–66.5 years) compared to those with the CC genotype (58.2 years, 95% CI, 55.6–60.7 years, *P* = 0.016). Few women had the GG genotype, but all three women with this genotype were young (aged 23, 33, and 35 years).

**Table 1:** **Characteristics of the 200 women with breast cancer relative to their rs11515 genotype**.

Characteristic	rs11515 genotype			Significance
	CC (*n* = 121)	CG (*n* = 76)	GG (*n* = 3)	
Age at surgery years	58.2 (55.6–60.7)	63.3 (60–66.5)	30.3 (14.1–46.5)	0.0001
Tumor grade				
1	17 (15.2%)	9 (11.8%)	3 (100%)	ns
2	42 (37.5%)	34 (44.7%)		
3	53 (47.3%)	33 (43.4%)		
Not known	9			
Lymph/vascular invasion present	37 (30.6%)	38 (50%)	1 (33.3%)	ns
Tumor size (mm)	24.2 (21–27.3)	26.4 (22.5–30.4)	38.3 (18.8–57.9)	ns
Metastases to lymph nodes				
None	73 (60.3%)	29 (38.2%)	1 (33.3%)	0.007
1 node	23 (19%)	13 (17.1%)	1 (33.3%)	
≥2 nodes	25 (20.7%)	34 (44.7%)	1 (33.3%)	
Estrogen Receptor				
Negative	34 (30.4%)	20 (26.3%)	1 (33.3%)	ns
Positive	72 (64.3%)	52 (68.4%)	2 (66.7%)	
Equivocal	6 (5.4%)	4 (5.3%)		
Data not known	10	0		
Progesterone receptor				
Negative	47 (42%)	31 (40.8%)	1 (33.3%)	ns
Positive	56 (50%)	37 (48.7%)	2 (66.7%)	
Equivocal	9 (8%)	8 (10.5%)		
Data not known	9	0		
HER2 receptor				
Negative	30 (58.8%)	24 (75%)	2 (66.7%)	ns
Positive	16 (31.4%)	8 (25%)	1 (33.3%)	
Equivocal	5 (9.8%)	0		
Data not known	69	44		

*Results are the mean (95% confidence intervals), or the *n* value (percent positive)*.

**P* values represent the comparison between three genotypes*.

*ns, not significant*.

The CG genotype was associated with a more aggressive tumor with greater lymph node involvement. Forty-five percent of women with the CG genotype had two or more nodes with malignant cells, and 38% had no nodal involvement. In those with the CC genotype, 21% had two or more nodes with malignant cells and 60% had no nodal involvement (*P* = 0.007). The tumor size and hormone receptor statuses did not differ between the rs11515 genotypes.

The rs11515 polymorphism is located in close proximity to another polymorphism associated with breast cancer (rs3088440) (34). To determine if rs3088440 genotypes were increased in the breast cancer cohort, the rs3088440 polymorphism was genotyped. In the breast cancer, cohort 78% (*n* = 155) were homozygote for the major allele, 21% were heterozygote (*n* = 42), and 1.5% (*n* = 3) were homozygote for the minor allele. In the control cohort, 82% (*n* = 164) were homozygote for the major allele, 17% were heterozygote (*n* = 34), and 1% (*n* = 2) were homozygote for the minor allele. The rs3088440 genotype was not significantly different in women with breast cancer compared to the control population. Nine individuals were heterozygote for both the rs11515 and rs3088440 SNPs.

### The CG Genotype Was Associated With Increased *ANRIL* Expression in Breast Tumors

In glioblastoma patients, the CG genotype was associated with increased loss of *p16^*INK4a*^* and *p14^*ARF*^* due to gene deletions at 9p21 (17). To determine if this was the case in breast cancer, DNA was isolated from 25 breast tumors (12 from women with the CG genotype and 13 with the CC genotype), and multiplex PCR used to estimate the gene dosage of *CDKN2A* exon 1a (*p16^*INK4a*^*) and exon 1b (*p14^*ARF*^*) relative to an internal *beta-globin* gene fragment (29). All breast tumors had retained exon 1a and exon 1b (data not shown), suggesting the CG genotype was not associated with loss of *p16^*INK4a*^* and *p14^*ARF*^* gene dosage.

Polymorphisms in *CDKN2A*/*2B*/*ANRIL* may regulate gene expression within the cluster. To test this hypothesis, we investigated associations between rs11515 genotypes and expression of *CDKN2A* (transcripts, *p16^*INK4a*^* and *p14^*ARF*^*), and *CDKN2B* in 25 breast tumors (12 from women with the CG genotype and 13 with the CC genotype). Due to the limited availability of tumor tissue for those with the CG genotype, *ANRIL* was measured in 21 breast tumors (8 from women with the CG genotype and 13 with the CC genotype).

**Figure 1:**
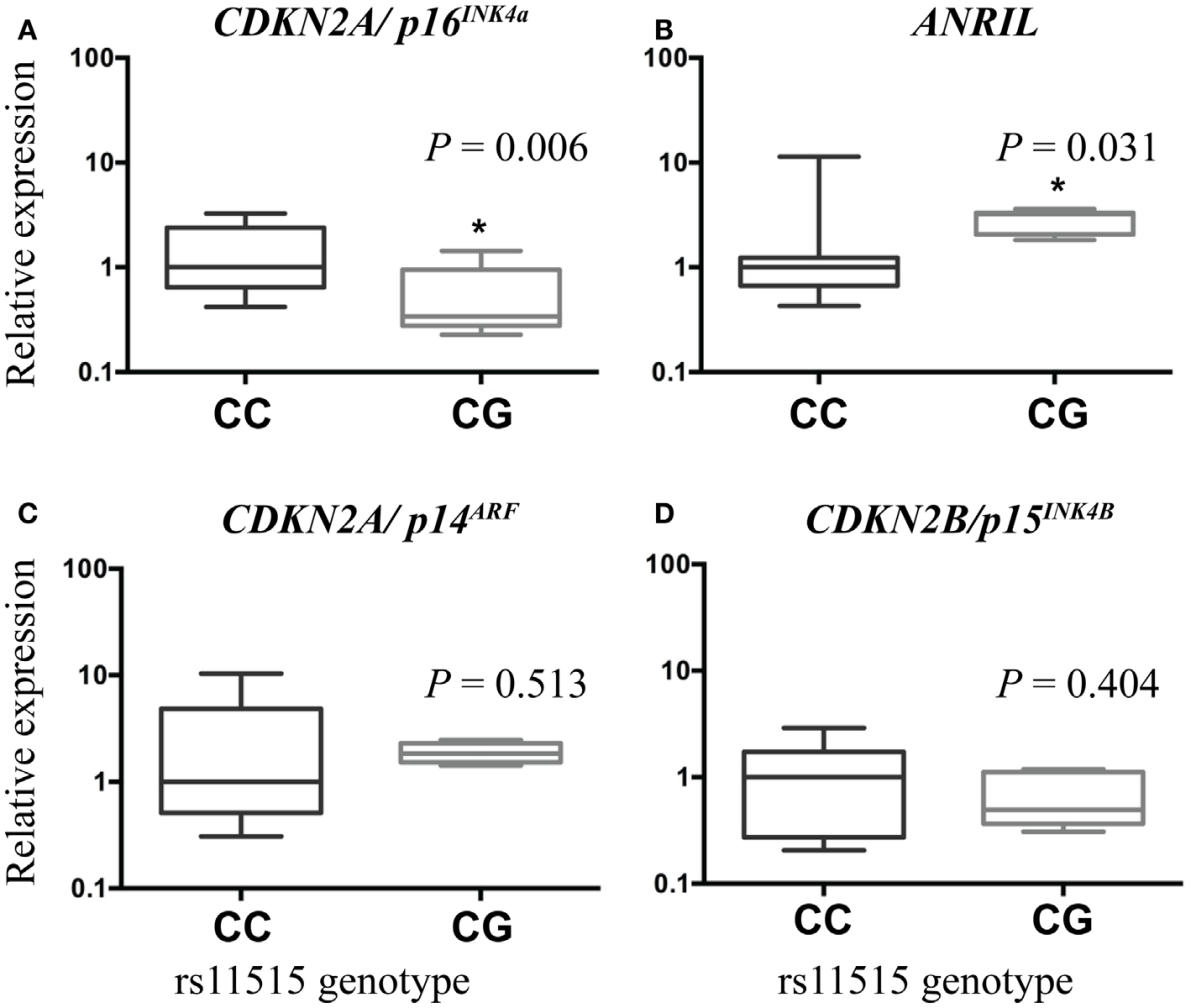
**Boxplots illustrating associations between gene expression and rs11515 genotypes in breast tumor samples**. Compared with tumors with a CC genotype, expression of *CDKN2A/p16^INK4a^* was lower **(A)** and expression of *ANRIL* was higher **(B)** in tumors with a CG genotype. There were no associations between rs11515 genotype and expression levels of *CDKN2A/p14^ARF^* **(C)** or *CDKN2B/p15^INK4b^* **(D)** between CG and CC genotypes in breast tumor tissue.

Compared to the CC genotype, the CG genotype was associated with lower expression of *CDKN2A/p16^*INK4a*^* [2.6-fold, *P* = 0.006 (adjusted for age), Figure 1A] and higher expression of *ANRIL* (2.3-fold, *P* = 0.031, Figure 1B). There was no difference in expression of *CDKN2A/p14^*ARF*^* (*P* = 0.513, Figure 1C) or *CDKN2B* [*P* = 0.404 (adjusted for age), Figure 1D] between the genotype groups.

**Figure 2:**
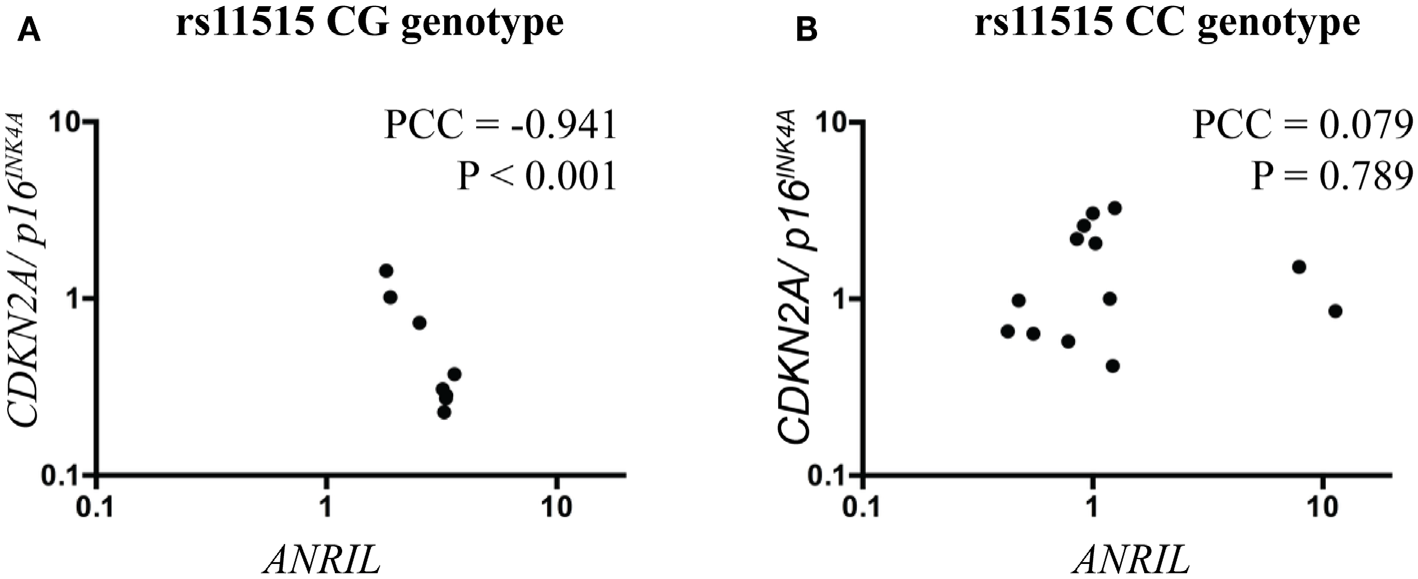
**Scatterplots illustrating the interaction between rs11515 genotypes and *ANRIL* on *CDKN2A/p16^INK4a^* gene expression in breast tumor samples**. **(A)** In tumors with a CG genotype, *ANRIL* and *CDKN2A/p16^INK4a^* levels were strongly negatively correlated. **(B)** In contrast, in tumors with a CC genotype, *ANRIL* and *CDKN2A/p16^INK4a^* levels were not correlated. PCC = Pearson correlation coefficient.

Because *ANRIL* may regulate the expression of *CDKN2A/p16^*INK4a*^* and because expression levels of both of these genes were altered in association with the rs11515 genotype, we investigated the relationship between rs11515 genotype, *ANRIL*, and *CDKN2A/p16^*INK4a*^* expression further, using analysis of covariance. We identified a significant interaction between rs11515 and *ANRIL* on expression of *CDKN2A/p16^*INK4a*^*: in patients with a CG genotype levels of *ANRIL* were strongly negatively correlated with levels of *CDKN2A/p16^*INK4a*^* (Pearson Correlation Coefficient = −0.941, *P* < 0.001, Figure 2A), whereas in CC patients there was no correlation between *ANRIL* and *CDKN2A/p16^*INK4a*^* (Pearson Correlation Coefficient = 0.079, *P* = 0.798, Figure 2B). The interaction between rs11515 and *ANRIL* on *CDKN2A/p16^*INK4a*^* expression remained significant after adjustment for age (adjusted *P* = 0.014) indicating that the relationship between rs11515, *ANRIL*, and *CDKN2A/p16^*INK4a*^* was not influenced by this potential confounding factor.

**Figure 3:**
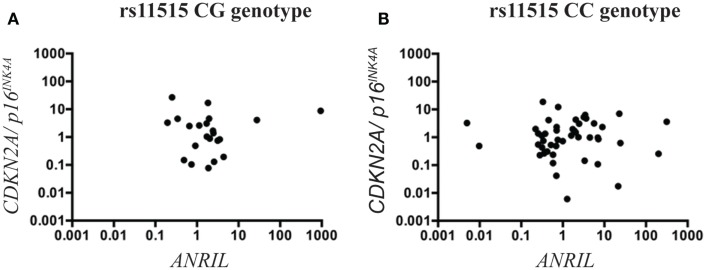
**Scatterplots illustrating the interaction between rs11515 genotypes and *ANRIL* on *CDKN2A/p16^INK4a^* gene expression in heart tissue**. The CG genotype **(A)** and the CC **(B)** genotype in heart tissue showed was no correlation between *ANRIL* and *CDKN2A/p16^INK4a^* expression levels.

To test whether the association between rs11515 genotypes and altered expression of the *CDKN2A*/*2B*/*ANRIL* cluster occurred in other tissues, we investigated rs11515 genotypes in 108 heart donors. The rs11515 genotype frequencies in heart donors were CC 67.6% (*n* = 73), CG 28.7% (*n* = 31), and GG 3.7% (*n* = 4) and were in Hardy–Weinberg equilibrium (*P* = 0.755). There were no associations between rs11515 genotypes and age and left ventricular ejection fraction and cause of death (Table 2). There were also no associations between rs11515 genotype and expression of *CDKN2A*/*p16^*INK4a*^* (*P* = 0.940), *CDKN2A/p14^*ARF*^* (*P* = 0.641), *CDKN2B* (*P* = 0.116), or *ANRIL* (*P* = 0.701) in myocardium. No interaction between the rs11515 genotype and *ANRIL* on *CDKN2A/p16^*INK4a*^* levels (*P* = 0.124) was identified. In heart donors, there was no significant interaction between rs11515 genotypes and *ANRIL* on expression of *CDKN2A/p16^*INKa*^*, Figure 3. All myocardial gene expression analyses were adjusted for age and gender. These results suggest the effect of rs11515 genotypes on 9p21 gene expression is tissue and or disease related.

**Table 2:** **Characteristics of the 108 heart donors relative to their rs11515 genotype**.

Characteristic	rs11515 genotype			Significance
	CC (*n* = 73)	CG (*n* = 31)	GG (*n* = 4)	
Age (years)	47.9 (45.0–50.9)	46.5 (41.9–51.0)	57.0 (44.5–69.5)	0.294
Gender				
Male	41 (56.2%)	12 (40.0%)	2 (50.0%)	0.328
Female	32 (43.8%)	18 (60.0%)	2 (50.0%)	
Left ventricular ejection fraction (%)	53.0 (48.1–57.9)	49.4 (41.6–57.2)	54.2 (36.1–72.2)	0.722
Cause of death				
Cerebral vascular accident	52 (71.2%)	22 (73.3%)	4 (100.0%)	0.986
Gun shot wound	7 (9.6%)	3 (10.0%)	0 (0.0%)	
Motor vehicle accident	7 (9.6%)	2 (6.7%)	0 (0.0%)	
Head trauma	5 (6.8%)	2 (6.7%)	0 (0.0%)	
Anoxia	2 (2.7%)	1 (3.3%)	0 (0.0%)	

*Results are the mean (95% confidence intervals) or the *n* value (percent positive). *P* values represent the comparison between three genotypes*.

## Discussion

The current study adds breast cancer to a growing list of malignancies associated with the rs11515 minor allele (10–17). The CG genotype was more frequent in those with breast cancer, associated with an older age and a more invasive tumor, and found with a negative correlation between *ANRIL* and *p16^*INK4a*^*. These data suggest the CG genotype is associated with a more aggressive tumor.

Reduced p16^INK4a^ tumor suppressor function in combination with increased *ANRIL* offers an explanation for increased cancer susceptibility with the rs11515 minor allele. An association between the CG genotype and lower expression of *p16^*INK4a*^* is consistent with previous studies. In melanoma and colorectal carcinoma, the G allele was associated with loss of *p16^*INK4a*^* and *p14^*ARF*^* expression, and in glioblastoma, CG genotype was associated with reduced *p16^*INK4a*^* and *p14^*ARF*^* gene dosage (13, 14, 17). The observation that *ANRIL* levels were higher and negatively correlated with *p16^*INK4a*^* in patients with the CG genotype, suggests *ANRIL* may be upregulated and acting to repress *p16^*INK4a*^* expression. There is evidence to suggest *ANRIL* affects expression of 9p21 transcripts by acting as a scaffold to guide chromatin-remodeling proteins toward 9p21 (19, 21, 35–37). *ANRIL* may promote the formation of heterochromatin facilitating reduced expression of *p16^*INK4a*^* expression. A specific example is the binding of *ANRIL* to polycomb repressive complexes 1 and 2 (PRC1 and PRC2) that is thought to direct epigenetic regulation and reduced *p16^*INK4a*^* and *p15^*INK4b*^* (19, 35).

**Figure 4:**
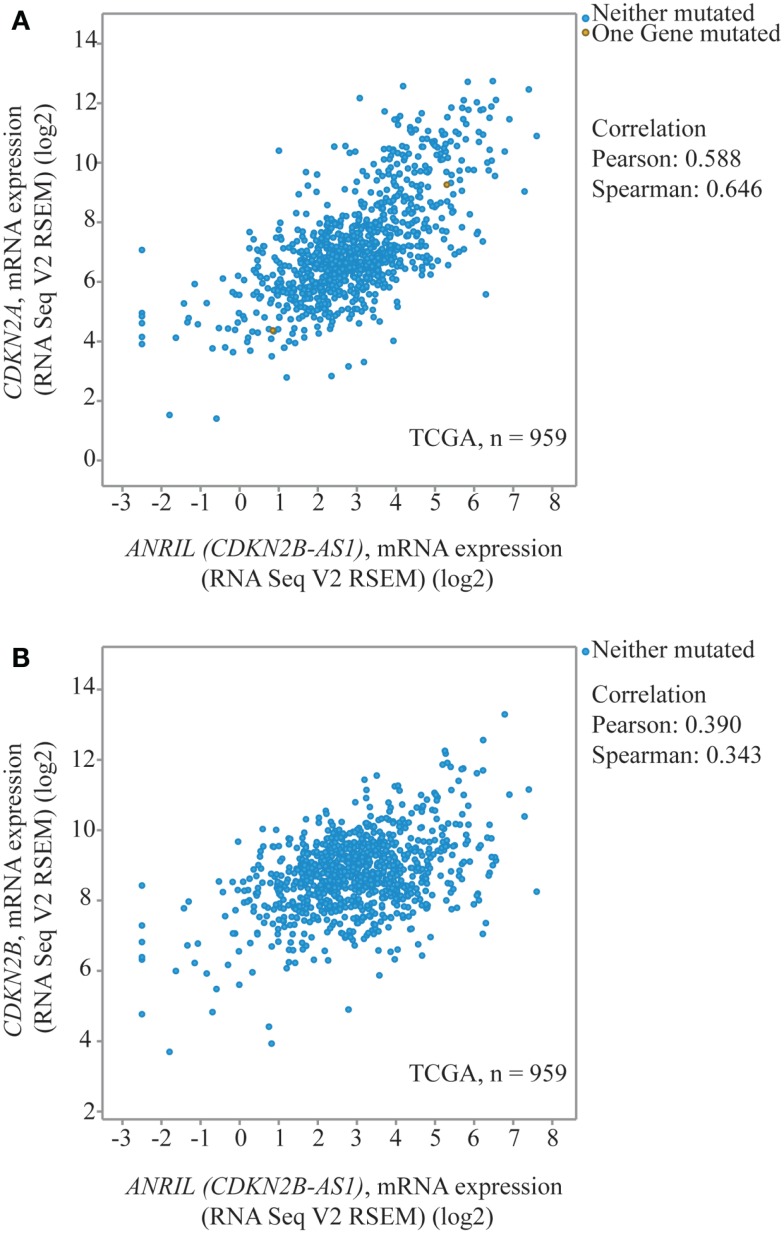
**Dot-plots from TCGA data for invasive breast cancer to illustrate correlations between 9p21 transcripts**. *ANRIL* (*CDKN2B-AS1*) and *CDKN2A* (*p16^INK4a^* and *p14^ARF^*) **(A)** and *CDKN2B* (*p15^INK4b^*) **(B)** (38, 39).

Other data suggest increased *ANRIL* would be associated with increased rather than decreased expression at 9p21 in breast cancer. Using TCGA expression data for invasive breast cancer, *ANRIL* and *CDKN2A (p14^*ARF*^* and *p16^*INK4a*^* combined) were positively correlated (Pearson correlation 0.588, Figure 4A) (38, 39), with a marginal positive correlation between *ANRIL* and *p15^*INK4b*^* (Pearson Correlation 0.39, Figure 4B). An analysis of 12 breast tumors by Pasmant et al. (18) found increased *ANRIL* correlated with increased *p14^*ARF*^*, *p16^*INK4a*^*, and *p15^*INK4b*^* (18). The available TCGA data are not divided based on the rs11515 genotype. Considering most women will be homozygote for the major rs11515 allele, the inconsistency between the TCGA data with that from the current study raises the possibility that the relationship between *ANRIL* and *p16^*INK4a*^* may be influenced by the rs11515 genotype, with an inverse relationship for those with the CG but not the CC genotype.

Although we argue that the CG genotype is correlated with increased risk toward breast cancer due to increased nodal involvement, increased *ANRIL*, and reduced *p16^*INK4a*^* expression, it could be argued that the CC genotype is the risk genotype. Patients with the CC genotype were younger. In another study, increased rather than decreased expression of *p16^*INK4a*^* was associated with a higher breast tumor grade and increased proliferative capacity (40). It could be that the CG genotype protects against tumors initially, but with age tumor surveillance may be compromised leading to a more aggressive tumor. The current study did not investigate *ANRIL* expression in normal associated breast tissue, so we did not determine whether it is the CG or CC genotype that is aberrant for *ANRIL* expression in breast tumors. Those with the GG genotype were young and this supports the G rs11515 allele being the risk allele for breast cancer. However, few had the GG genotype and breast tissue from GG homozygotes was not available to determine if *ANRIL* expression is greatest in these women.

The association between the CG genotype and *ANRIL* did not occur in heart tissue, suggesting the effect of rs11515 alleles is tissue or disease specific. Multiple isoforms of *ANRIL* exist including both linear and circular forms with tissue-specific expression patterns identified (41, 42). *ANRIL* isoforms with an ALU motif were found to be more effective in recruiting chromatin-remodeling proteins (21). The specific *ANRIL* isoforms increased in breast tumors were not identified so the tissue-specific differences between breast cancer and myocardium could be attributed to different *ANRIL* isoforms being present. The rs11515 SNP is predicted to affect the binding site of microRNA-601 (43), so the effect of rs11515 genotypes may be dependent of the expression of other transcripts.

Other 9p21 polymorphisms have been correlated with cancer including breast cancer (rs1011970), glioma (rs1063192, rs2157719, rs1412829, and rs4977756), basal cell carcinoma (rs2151280), nasopharyngeal carcinoma (rs1412829), and breast cancer (rs1011970 and rs3088440) [reviewed in Ref. (34, 44)]. Consequently, another polymorphism in linkage with rs11515 could be the causal allele for the increased *ANRIL* expression. The rs3088440 polymorphism is located 40 bp downstream of rs11515. In a large cohort of over 3000 women with breast cancer, the rs3088440 minor allele was associated with breast cancer (34), and in pancreatic cancer associated with a reduced time to tumor progression and a poorer response to therapy (45). The rs3088440 genotypes were not significantly increased in the breast cancer cohort in the current study; however, a limitation of the current study was a smaller cohort was used (34). The majority of individuals’ heterozygote for the rs11515 allele were homozygote for the rs3088440 major allele; therefore, it is possible that a haplotype contributes to the associations found in this study instead of the rs11515 genotype alone.

## Conclusion

To our knowledge, this is the first report of an association between the rs11515 minor allele with breast cancer and a negative correlation between *ANRIL* and *p16^*INK4a*^* levels with the CG genotype. Reduced expression of *p16^*INK4a*^* may be one mechanism by which the rs11515 CG genotype increases cancer risk.

## Author Contributions

JR – designed and supervised experimental studies, and edited the manuscript; AP – performed PCR, data analysis and interpretation, wrote, and edited the manuscript; AA – performed PCR, analyzed data, and edited the manuscript; HM, MC, CM, WS, WHWT – designed research, interpreted data, and edited the manuscript. CF – performed the statistical analyses, interpreted data, and edited the manuscript; IAR – interpreted TCGA data, designed research, and edited the manuscript; NA – conceived the study, interpreted data, and edited the manuscript. TS – conceived the study, performed PCR, designed research, and wrote and edited the manuscript.

## Conflict of Interest Statement

The authors declare that the research was conducted in the absence of any commercial or financial relationships that could be construed as a potential conflict of interest.
